# Silencing of Long Non-coding RNA SMAD5-AS1 Reverses Epithelial Mesenchymal Transition in Nasopharyngeal Carcinoma via microRNA-195-Dependent Inhibition of SMAD5

**DOI:** 10.3389/fonc.2019.01246

**Published:** 2019-12-13

**Authors:** Siwei Li, Bo Zhao, Haiying Zhao, Cui Shang, Man Zhang, Xiaoxia Xiong, Jinjin Pu, Bohua Kuang, Guangrui Deng

**Affiliations:** ^1^Department of Oncology, Tongji Huangzhou Hospital, Huazhong University of Science and Technology, Huanggang, China; ^2^Department of Radiation Oncology, The Affiliated Hospital of Guilin Medical University, Guilin, China; ^3^Graduate School, Guillin Medical University, Guilin, China; ^4^Cancer Center, Union Hospital, Tongji Medical College, Huazhong University of Science and Technology, Wuhan, China; ^5^State Key Laboratory of Oncology in South China, Department of Experimental Research, Sun Yat-sen University Cancer Center, Collaborative Innovation Center for Cancer Medicine, Guangzhou, China

**Keywords:** nasopharyngeal carcinoma, SMAD5-AS1, microRNA-195, SMAD5, epithelial-mesenchymal transition, migration, invasion, apoptosis

## Abstract

Long non-coding RNAs (lncRNAs) have gained widespread attention in recent years as a key regulator of diverse biological processes, but the knowledge of the mechanisms by which they act is still very limited. Differentially expressed lncRNA SMAD5 antisense RNA 1 (SMAD5-AS1) in nasopharyngeal carcinoma (NPC) and normal samples shown by *in silico* analyses were selected as the main subject, and then microRNA-195 (miR-195) was suggested to bind to SMAD5-AS1 and SMAD5. Therefore, the purpose of the present study was to investigate the effects of SMAD5-AS1/miR-195/SMAD5 on epithelial-mesenchymal transition (EMT) in NPC cells. High expression of SMAD5-AS1 and SMAD5 but low miR-195 expression was determined in NPC tissues and NPC cell lines by RT-qPCR and western blot analysis. SMAD5-AS1 could upregulate SMAD5 expression by competitively binding to miR-195 in NPC cells. Loss- and gain-of-function investigations were subsequently conducted in NPC cells (CNE-2 and CNE-1) to explore the role of SMAD5-AS, miR-195 and SMAD5 in NPC progression by assessing cellular biological functions and tumorigenic ability *in vivo* as well as determining the expression of EMT markers. Downregulation of SMAD5-AS1 or SMAD5 or overexpression of miR-195 led to inhibited NPC cell proliferation, invasion and migration and reversed EMT, enhanced apoptosis *in vitro* as well as restrained tumor growth *in vivo*. In conclusion, our findings indicate that silencing of lncRNA SMAD5-AS1 induces the downregulation of SMAD5 by miR-195, eventually repressing EMT in NPC. Hence, SMAD5-AS1 may represent a potential therapeutic target for NPC intervention.

## Introduction

Nasopharyngeal carcinoma (NPC) had been estimated to reach 42,100 new morbidity and 21,320 mortality in 2013, accounting for ~1.14% of total cancer cases and 0.96% of total cancer-related mortality that year in China (1). Although intensity-modulated radiation therapy (IMRT) and radio-chemotherapy have significantly enhanced decentralized control of NPC, the clinical treatment is not satisfactory enough (2). Distant metastasis remains a primary reason for failure of the treatment (3). It has been reported that epithelial-mesenchymal transition (EMT) plays a crucial role in metastasis of malignancies (4). Several studies have strongly implicated the activation of long non-coding RNAs (lncRNAs) in the tumorigenesis and progression of NPC, such as n326322 and ROR (5, 6).

LncRNAs have emerged as key components of the address code, allowing protein complexes, genes, and chromosomes to be trafficked to proper position and complete appropriate activation and deactivation (7). Recently, lncRNAs have been demonstrated to play vital roles in some cancers, especially in NPC prior to or during the process of invasion-metastasis (8). LncRNA H19 has been found to participate in the metastasis of NPC through upregulating EZH2 expression in tumor tissues (9). *In silico* analysis shows a regulatory relationship between lncRNA SMAD5 antisense RNA 1 (SMAD5-AS1) and microRNA-195 (miR-195). miR-195, one of the miR-16/15/195/424/497 family members, has been indicated to be a tumor inhibitor that plays a crucial mediatory role in tumorigenesis (10). Recently, it has been reported that miR-195 is underexpressed in bladder cancer (11). In addition, based on target gene prediction software, a target of miR-195, SMAD family member 5 (SMAD5), was identified in this study. As a group of intracellular proteins, SMADs are involved in transforming growth factor beta (TGF-β) signaling in cancer progression and metastasis (12). A previous study has suggested that SMAD2 signaling is involved in the inhibition of EMT in NPC cells (13). Moreover, miR-145 overexpression has been found to inhibit invasion and metastasis in NPC cells by targeting SMAD3 (14). We speculated that SMAD5-AS1, SMAD5, and miR-195 might be interacted during cancer progression and their regulatory relationship could be potentially implicated in NPC. In this study, we aimed to investigate the potential effect of SMAD5-AS1, SMAD5, and miR-195 in EMT of NPC cells and to explore the underlying mechanisms.

## Materials and Methods

### Ethics Statement

This study was approved by the Ethics Committee of Tongji Huangzhou Hospital, Huazhong University of Science and Technology. Informed written consent was obtained from each patient prior to the study. Animal experiments were conducted in strict accordance with the Guide for the Care and Use of Laboratory animals published by the US National Institutes of Health (NIH). Great efforts were made to minimize the number of animals used in the experiments and their suffering.

### *In silico* Analysis

Expression datasets of NPC were obtained from the Gene Expression Omnibus (GEO, https://www.ncbi.nlm.nih.gov/geo/) database. The “limma” package of the R programming language was used for differential analysis of the results obtained from these datasets, and the “pheatmap” package was employed to construct heat maps of differentially expressed genes (DEGs). A Venn Diagram was obtained using online Calculate and draw custom Venn Diagrams (http://bioinformatics.psb.ugent.be/webtools/Venn/). The cellular sublocalization of SMAD5-AS1 was predicted in the lncATLAS database (http://lncatlas.crg.eu/). Then, the RNA22 database (https://cm.jefferson.edu/rna22/) and miRcode database (http://www.mircode.org/) were used to predict the downstream miRs of SMAD5-AS1, and the downstream target genes of miR-195 were predicted by the miRDB database (http://mirdb.org/miRDB/index.html), mirDIP database (http://ophid.utoronto.ca/mirDIP/index.jsp#r), TargetScan database (http://www.targetscan.org/vert_71/), DIANA database (http://diana.imis.athena-innovation.gr/DianaTools/index.php?r=microT_CDS/index), and starBase database (http://starbase.sysu.edu.cn/). The SMAD5-related signaling pathways were retrieved using the Kyoto Encyclopedia of Genes and Genomes (KEGG) database (https://www.kegg.jp/kegg/pathway.html).

### Study Subjects

A total of 41 patients with NPC at the Tongji Huangzhou Hospital, Huazhong University of Science and Technology from April 2016 to April 2017 were enrolled in the study. All the enrolled cases were pathologically diagnosed as poorly differentiated squamous cell carcinoma. Among them, 28 were males and 13 were females, with a median age of 46 years. None of them were not complicated with other tumors and received any treatment before admission. There were 2 patients in stage I, 10 patients in stage II, 17 patients in stage III, and 12 patients in stage IV (2). NPC tissues were taken as an experimental group and adjacent normal tissues 5 cm away from the lesions as a control group. These tissues were preserved at −80°C for following experiments. The expression of SMAD5-AS1, miR-195, and SMAD5 in NPC tissues and adjacent normal tissues was detected using reverse transcription quantitative polymerase chain reaction (RT-qPCR), western blot analysis and immunohistochemistry.

### Cell Culture

The 5-8F NPC cell line with high tumorigenicity and high metastatic potential, 6-10B NPC cell line without metastatic potential, highly differentiated nasopharyngeal squamous carcinoma cell line CNE-1, poorly differentiated nasopharyngeal squamous carcinoma cell lines CNE-2 and HONE-1, and immortalized nasopharyngeal epithelial cells NP69 were stored at the Tongji Huangzhou Hospital, Huazhong University of Science and Technology. Cells were cultured in Roswell Park Memorial Institute (RPMI) 1640 complete medium (Thermo Scientific, Waltham, MA, USA) containing 10% fetal bovine serum (FBS; GIBCO, Grand Island, N.Y., USA) and incubated at 37°C with 5% CO_2_. Cell morphology was then observed using an inverted microscope (Olympus Deutschland GmbH, Hamburg, Germany) every day and passaged every 2–3 days.

### Cell Infection

Lentivirus was constructed for cell infection. Cells were assigned into short hairpin RNA (sh)-negative control (NC) group (cells infected with lentivirus carrying sh-NC), sh-SMAD5-AS1 group (cells infected with lentivirus carrying sh-SMAD5-AS1), mimic NC group (cells infected with lentivirus carrying mimic NC), miR-195 mimic group (cells infected with lentivirus carrying miR-195 mimic), sh-SMAD5 group (cells infected with lentivirus carrying sh-SMAD5), miR-195 mimic + overexpressed (oe)-NC group (cells infected with lentivirus carrying miR-195 mimic and oe-NC) and miR-195 mimic + oe-SMAD5 group (cells infected with lentivirus carrying miR-195 mimic and oe-SMAD5 plasmid). After cleavage with two endonucleases EcoRI and BamHI, fragments containing overexpressed and shRNAs and target vectors were inserted into pLVX-IRES-ZsGreenl vector and transformed into DH5α cells. Lentiviral supernatants were produced by infection into 293T cells with the helper plasmids pSPAX2 and pMD2G, and the supernatants were collected 48–72 h post-infection. The lentiviruses with a concentration higher than 10^7^ TU/mL were packaged and stored at −80°C for subsequent experiments. The supernatant containing different lentiviruses was dripped into CNE-2 cells upon 70–80% confluence. The medium was changed after 24 h, and the infection efficiency was tested after 48 h for subsequent experiments.

### RT-qPCR

Total RNA was extracted by Trizol (15596026, Invitrogen, Car, Cal, USA), and the concentration and purity of RNA were measured by Nano-Drop ND-1000 spectrophotometer. According to the instructions of PrimeScript RT reagent Kit (RR047A, Takara, Tokyo, Japan), RNA was reversely transcribed into complementary DNA (cDNA). The primers (Table 1) of SMAD5-AS1, miR-195, SMAD5, E-cadherin, Vimentin, U6, and glyceraldehyde 3-phosphate dehydrogenase (GADPH) were synthesized by Sangon Biotech Co., Ltd. (Shanghai, China). A total of 10 ng miRNA was reversely transcribed into cDNA using TaqMan® MicroRNA Reverse Transcription Kit (Applied Biosystems, Foster City, CA, USA). Real time quantitative PCR was performed to determine the expression of miRNA with TaqMan MicroRNA Assay (Applied Biosystems, Foster City, CA, USA). Real time fluorescence quantitative PCR was performed for the other RNAs using a SYBR®Premix Ex TaqTM II reagent kit (Takara Biotechnology Ltd., Dalian, Liaoning, China) on an ABI7500 real-time fluorescence quantitative PCR system (7500, ABI Company, Oyster Bay, NY, USA). With U6 as the internal control for miR-195 and GAPDH for the remaining genes, the fold changes of expression level were calculated by 2^−ΔΔ*Ct*^ method with the following formula: ΔCt = CT_(targetgene)_–CT_(internalcontrol)_, ΔΔCT = ΔCt_(experimentalgroup)_ − ΔCt_(controlgroup)_.

**Table 1:** Primer sequences for RT-qPCR.

**Gene**	**Primer sequences**
SMAD5-AS1	F: 5′-GACGCTGCTTTGGCATTCTC-3′
	R: 5′-TGCTTCTATGCTGGGTGACTC-3′
miR-195	F: 5′-AGCTTCCCTGGCTCTAGCA-3′
	R: 5′-CTGGAGCAGCACAGCCAATA-3′
SMAD5	F: 5′-CCAGCAGTAAAGCGATTGTTGG-3′
	R: 5′-GGGGTAAGCCTTTTCTGTGAG-3′
E-cadherin	F: 5′-CGAGAGCTACACGTTCACGG-3′
	R: 5′-GGGTGTCGAGGGAAAAATAGG-3′
Vimentin	F: 5′-GACGCCATCAACACCGAGTT-3′
	R: 5′-CTTTGTCGTTGGTTAGCTGGT-3′
GADPH	F: 5′-GGAGCGAGATCCCTCCAAAAT-3′
	R: 5′-GGCTGTTGTCATACTTCTCATGG-3′
U6	F: 5′-CTCGCTTCGGCAGCACA-3′
	R: 5′-AACGCTTCACGAATTTGCGT-3′

*RT-qPCR, reverse transcription quantitative polymerase chain reaction; SMAD5, drosophila mothers against decapentaplegic protein; SMAD5-AS1, drosophila mothers against decapentaplegic protein antisense RNA 1; miR-195, microRNA-195; GADPH, glyceraldehyde 3-phosphate dehydrogenase; F, forward; R, reverse*.

### Immunohistochemistry

The expression of SMAD5 was detected by immunohistochemical streptavidin-perosidase (SP) kits (ab64261, Abcam Inc., Cambridge, UK). Briefly, NPC and adjacent normal tissues were fixed by 10% formalin, paraffin embedded, and then sectioned. The blocked sections were then incubated with primary antibody of rabbit monoclonal antibody to SMAD5 (1:50, ab40771, Abcam Inc., Cambridge, UK). Goat anti-rabbit immunoglobulin G (IgG) (1:500, ab97051, Abcam Inc., Cambridge, UK) was applied as secondary antibody. SMAD5 protein was predominantly expressed in the cytoplasm, and brown granules were visualized in the SMAD5-positive cells after staining. Images were captured and the positive rates were counted by Image J software.

### Dual Luciferase Reporter Gene Assay

The miR target gene prediction software was employed to predict the potential relationships among SMAD5-AS1, SMAD5, and miR-195. SMAD5-AS1 sequence and SMAD5-3'untranslated region (3'UTR) sequence containing binding sites were synthesized, namely SMAD5-AS1-wild type (WT) and SMAD5-WT, respectively. The mutation sequences SMAD5-AS1-mutnat (MUT) and SMAD5-MUT) were obtained by site-directed mutagenesis using QuikChange II®Site-Directed Mutagenesis kit (Stratagene, La Jolla, CA, USA). After endonuclease cleavage, the synthesized gene fragments were inserted into pmiR-RB-REPORT™ vector (Guangzhou RiboBio Co., Ltd., Guangzhou, China) to generate pmiR-RB-REPORT-SMAD5-AS1-WT, pmiR-RB-REPORT-SMAD5-WT, pmiR-RB-REPORT-SMAD5-AS1-MUT, and pmiR-RB-REPORT-SMAD5-MUT recombinant plasmids. The recombinant plasmids were, respectively, co-transfected with the miR-195 mimic plasmid or mimic control plasmid into human embryonic kidney (HEK)-293T cells. After 48 h of transfection, the cells were lysed, and the luciferase activity was detected using luciferase detection kits (RG005, Beyotime Biotechnology Co., Shanghai, China).

The detection of SMAD5 promoter transcriptional activity was then conducted. Briefly, the promoter sequence and full sequence of SMAD5 were obtained from the NCBI database (http://www.ncbi.nlm.nih.gov/gene). SMAD5 promoter region sequence was cloned into the pmirGLO vector (Promega Corporation, Madison, WI, USA) to construct pmirGLO-SMAD5 prom WT (SMAD5-promoter WT) vector. The SMAD5-promoter WT recombinant plasmid was co-transfected into HEK-293T cells with oe-SMAD5-AS1/sh-SMAD5-AS1 or oe-NC/sh-NC. The renilla luciferase expression vector pRL-TK (TaKaRa, Dalian, China) was taken as an internal reference. After the cells were cultured for 24 h, the luciferase activity was detected using the Dual-Luciferase Reporter Assay System (Promega, Madison, WI, USA).

### RNA Binding Protein Immunoprecipitation (RIP) Assay

CNE-2 cells were collected and washed with precooled phosphate buffer saline (PBS). Then, cells were lysed with RIP lysis buffer (P0013B, Beyotime Biotechnology Co., Shanghai, China) and ice-bathed for 5 min. RIP kit (Merck Millipore, Billerica, MA, USA) was employed to detect the binding SMAD5-AS1 to Argonaute2 (AGO2) protein. Magnetic beads in lysis buffer were incubated with 5 μg rabbit anti-human AGO2 antibody (ab186733, 1:50, Abcam, Shanghai, China) for 6 h at 4°C. After incubation, 100 μL cell lysate was added to the magnetic beads-antibody complex and incubated overnight at 4°C. Samples were placed on magnetic pedestals to collect magnetic beads-protein complexes. The samples were detached with proteinase K buffer to collect RNA, followed by RT-qPCR. The percentage of SMAD5-AS1 binding to AGO2 protein (%) was calculated with untreated cell supernatant as reference. Rabbit anti-human IgG (ab109489, 1:100, Abcam, Shanghai, China) was used as NC.

### RNA Pull-Down Assay

CNE-2 cells were treated with 50 nM biotin-labeled Bio-miR195-WT and Bio-miR195-MUT (Wuhan GeneCreate Biological Engineering Co., Ltd., Wuhan, China) for 48 h. The cells were collected and lysed in Pierce IP Lysis Buffer (Thermo Fisher Scientific, Waltham, MA, USA) for 10 min. The lysates were then incubated overnight at 4°C with Dynabeads® streptavidin beads (Thermo Fisher Scientific, Waltham, MA, USA), which were precoated with RNase-free bovine serum albumin (BSA) and yeast tRNA (TRNABAK-RO, Sigma-Aldrich, St. Louis, MO, USA). After incubation, the supernatant was obtained by magnetic separation. The miR-195-bound RNA was purified by Trizol, and RT-qPCR was applied to detect the enrichment of SMAD5-AS1.

### Fluorescence *in situ* Hybridization (FISH) Assay

Subcellular localization of SMAD5AS-1 in CNE-2 cells was determined using the FISH kit (C10910, Guangzhou RiboBio Co., LTD, Guangzhou, China). Cell slides were placed at the bottom of a 24-well plate, and cells were inoculated into the plate at a density of 6 × 10^4^ cells/well. When cell confluence reached 60–70%, culture medium was removed and cells were incubated at room temperature with 0.2 mol/L HCl and 0.3% Triton X-100, successively, and fixed at room temperature with 4% paraformaldehyde for 10 min. After each well was pre-hybridized with 20 μL RNA hybridization buffer at 55°C for 2 h, the cells in each well were incubated with RNA probe and hybridized overnight at 37°C in a wet box in the dark. After counterstaining using 4',6-diamidino-2-phenylindole (DAPI), the images were captured by a laser scanning confocal microscope (FV1000, Olympus Deutschland GmbH, Hamburg, Germany). The average optical density (OD) values of cells were measured by Image Pro-plus 6.0 software.

### 5-Ethynyl-2'-Deoxyuridine (EdU) Assay

EdU (10 uM, C0071L, Beyotime Biotechnology Co., Shanghai, China) assay was conducted to assess the cell proliferation. The cells were incubated with EdU solution at room temperature for 2 h, washed with PBS and then fixed with 4% paraformaldehyde for 15 min. After three times washing with PBS containing 3% BSA, the cells were incubated at room temperature with PBS containing 0.3% Triton X-100 for 10 min. Subsequently, with the addition of Click Additive Solution, the culture plate was incubated in the dark at room temperature for 30 min and washed with PBS containing 3% BSA three times. Following the addition of Hoechst 3334 reaction solution to culture plate, the plate was incubated (C1022, Beyotime Biotechnology Co., Shanghai, China) in the dark at room temperature for 10 min. The cells were observed under a fluorescence microscope. Three fields were selected to count the number of EdU stained cells (proliferative cells) and Hoechst 33342-stained cells (total cells). The proliferation rate of cells was calculated with the formula: cell proliferation rate = the number of proliferative cells/the number of total cells × 100%.

### Transwell Assay

NPC 5-8F cells in logarithmic growth phase were detached and resuspended with RPMI 1640 containing 1% FBS with the cell density adjusted to 5 × 10^4^ cells/100 μL. The apical chamber of each Transwell chamber was added with 100 μL cell suspension, and the basolateral chamber was added with 600 μL RPMI 1640 containing 10% FBS. The 24-well culture plate settled with cells in the Transwell chamber was cultured in saturated humidity with 5% CO_2_ at 37°C for 24 h. After removal of the culture medium of the apical chamber, the chamber was fixed in 4% paraformaldehyde at room temperature for 15 min. The liquid in the apical chamber was absorbed by dry cotton swabs, and the cells on the surface of the polyester film were gently wiped off using a cotton swab soaked in distilled water. The cells were stained in 1% crystal violet staining solution at room temperature for 15 min and washed by PBS for 2–3 times. Finally, images of each well were taken using a microscope with 5 visual fields randomly selected in each well.

Cell invasion was measured with pre-cooled Matrigel diluted with serum-free RPMI 1640 medium at a ratio of 1:8. Matrigel was evenly coated at the bottom membrane of Transwell chamber with a pre-cooled 200 μL pipette tip at 50 μL/well and placed in the incubator for 30 min for gelling. The other operations were similar to those used in the cell migration experiment.

### Flow Cytometry

Annexin V-fluorescein isothiocyanate (V-FITC) cell apoptosis detection kit (C1063, Beyotime Biotechnology Co., Shanghai, China) was used to detect the apoptosis of cells. After 24 h of transfection and culture, the CNE-2 cells were collected and resuspended in precooled PBS. After being centrifuged at 716 g for 5 min at room temperature, the cells were resuspended in precooled PBS and centrifuged at 716 g for 5–10 min. After being added with 300 μL Binding Buffer, cells were suspended and incubated with 5 μL Annexin V-FITC at room temperature in the dark for 15 min, followed by the addition of 10 μL propidium iodide (PI) and incubation with ice bath in the dark for 10–20 min. Flow cytometer (Cube6, Partec, Mu^..^nster, Germany) was applied to detect cell apoptosis. FITC was detected when the excitation wavelength was 480 and 530 nm and PI was measured when the excitation wavelength was over 575 nm.

### Western Blot Analysis

Tissues or cells were treated with radioimmunoprecipitation assay (RIPA) lysis buffer (P0013B, Beyotime Biotechnology Co., Shanghai, China) containing phenylmethylsulphonyl fluoride (PMSF) and phosphatase inhibitors. The bicinchoninic acid (BCA) protein quantitative kit (Beyotime Biotechnology Co., Shanghai, China) was used to measure protein concentration. A total of 30 μg of protein was separated by sodium dodecyl sulfate-polyacrylamide gel electrophoresis (SDS-PAGE) and then transferred onto a nitrocellulose membrane. The membrane was then blocked with 5% skimmed milk in Tris-buffered saline with Tween 20 (TBST) for 1.5 h and then incubated overnight at 4°C with the following antibodies: rabbit monoclonal antibody to SMAD5 (1:2000; ab40771), rabbit monoclonal antibody to E-cadherin (1:1000, ab180960), rabbit monoclonal antibody to Vimentin (1:1000, ab103477), rabbit monoclonal antibody to BMP2 (1:1000, ab214821), rabbit monoclonal antibody to p-SMAD5 (1:1000, ab40771), mouse monoclonal antibody to B-cell lymphoma 2 (Bcl-2) (1:1000, sc-509, Santa Cruz Biotechnology, Inc., Santa Cruz, CA, USA), mouse monoclonal antibody to Bcl-2-associated X protein (Bax) (1:1000, sc23959, Santa Cruz Biotechnology, Inc., Santa Cruz, CA, USA), and rabbit polyclonal antibody to β-actin (1:5000, ab8227). All antibodies except the mouse monoclonal antibodies to Bax and Bcl-2 were purchased from Abcam, Inc. (Cambridge, UK). After three washes with TBST (15 min for each), the membrane was incubated with secondary antibody goat anti-rabbit IgG (1:2000 - 1:50000, ab205718, Abcam Inc., Cambridge, UK) and goat anti-mouse IgG (1:10000, Jackson ImmunoResearch Laboratories, West Grove, PA, USA) at room temperature for 2 h. The protein bands were developed using enhanced chemiluminescence (ECL) luminescent solution. Images were captured by SmartView Pro 2000 (UVCI-2100, Major Science Fermenters and Bioreactors Manufacturers Inc., CA, USA). Quanity One software was applied to analyze the gray level of protein bands.

### Xenograft Tumor in Nude Mice

A total of 48 BALB/c nude mice aged 4–6 weeks and weighing 18–22 g were purchased from Hunan SJA Laboratory Animal Co., Ltd. (http://www.hnsja.com/, Changsha, Hunan). The mice were housed in a specific pathogen free (SPF) laboratory at constant temperature (25°C ± 2°C) and constant humidity (45–50%) with 12 h of dark/light cycle. Mice were fed with autoclaved standard laboratory food and free to access sterile water. The nude mice were randomly grouped into sh-NC group, sh-SMAD5-AS group, mimic-NC group, miR-195 mimic group, sh-NC group, sh-SMAD5 group, miR-195 mimic + oe-NC group, and miR-195 mimic + oe-SMAD5 group by subcutaneous injection of the stably transfected cells into dorsal surface at the root of right hind limb in nude mice. The length and width of the tumors were measured with vernier calipers every 5 days and recorded as a and b, respectively. The tumor volume was calculated by the following formula: V = ab^2^/2. After 30 days, the nude mice were euthanized and the tumors were dissected. The final tumor weight was measured. The growth inhibition rate of tumors was calculated according to the following formula: the growth inhibition rate of tumor = (1—the weight of tumors in the experimental group/the weight of tumors in the control group) × 100%. The tumor specimens were stored at −80°C for subsequent detection of E-cadherin and Vimentin.

### Statistical Analysis

Statistical analyses were conducted by SPSS 21.0 (IBM Corp. Armonk, NY, USA). Measurement data were expressed as mean ± standard deviation. Paired *t*-test was performed for comparison between adjacent normal tissues and NPC tissues. Independent sample *t*-test was applied to compare the data between two groups. One-way analysis of variance (ANOVA) was employed to compare the data among multiple groups, followed by Tukey's *post-hoc* test. *p* < 0.05 indicates that the difference was statistically significant.

## Results

### The Functional Significance of SMAD5-AS1, miR-195, and SMAD5 Are Associated With NPC

**Figure 1:**
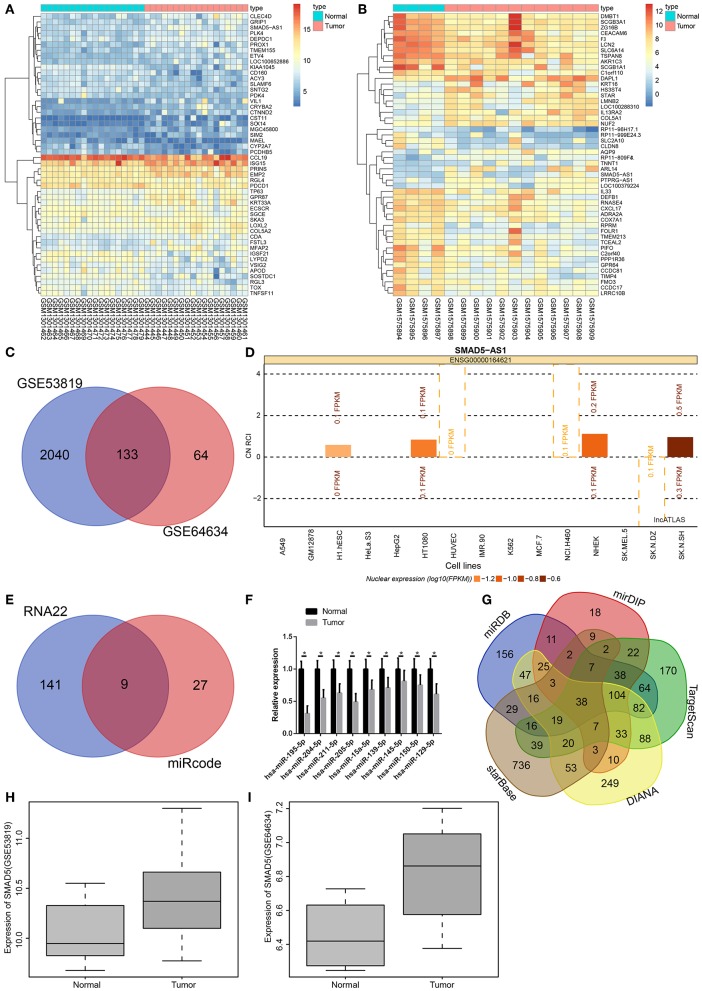
SMAD5-AS1, SMAD5, and miR-195 might be involved in NPC. **(A,B)** Heat maps of DEGs in NPC expression datasets GSE53819 and GSE64634, in which the abscissa represents the sample number, and the ordinate represents the DEGs; the dendrogram on the left refers to gene expression cluster; each rectangle corresponds to a sample expression value; the histogram at the upper right refers to color gradation. **(C)** Venn analysis of DEGs in NPC datasets, where the two circles represent the number of DEGs in the two datasets, and the middle part represents the intersection of two sets of data. **(D)** The expressing location of SMAD5-AS1 in cells. The abscissa represents different cell lines. The histogram above line 0 indicates that SMAD5-AS1 is expressed in the cytoplasm while histogram below line 0 shows that SMAD5-AS1 is mainly expressed in the nucleus. **(E)** The prediction of downstream regulatory miRNAs of SMAD5-AS1. The two circles represent the prediction results of RNA22 database and miRcode database, respectively, and the middle part represents the intersection of two clusters of data. **(F)** miRNA expression in NPC samples and adjacent normal samples determined by RT-qPCR. *p < 0.05 vs. the normal group. *n* = 20. **(G)** The predicted target genes of miR-195. Five irregular graphs, respectively, represent the prediction results in five databases, and the middle part represents the intersection of five sets of data. **(H,I)** The expression of SMAD5 in NPC based on the datasets GSE53819 and GSE64634, with sample type as the abscissa and gene expression as the ordinate. The expression of SMAD5 in normal samples is shown in the left box diagram, while the expression of SMAD5 in tumors is indicated in the right box diagram.

Microarray-based gene expression profiling was initially used to identify NPC-related DEGs. Two NPC expression datasets GSE53819 and GSE64634 were retrieved from the GEO database (https://www.ncbi.nlm.nih.gov/geo/). Through differential analysis of gene expression between NPC sample and adjacent normal samples, 2173 and 197 DEGs were selected from the two datasets, respectively. The expression heat maps of 50 DEGs in the two datasets are shown in Figures 1A,B, respectively. Next, Venn analysis was performed to obtain the overlapping DEGs in the two datasets (Figure 1C), which showed 133 intersected DEGs. Among the 133 DEGs, SMAD5-AS1 was found to be an upregulated gene in NPC. Microarray-based gene expression profiling also indicated that the expression of SMAD5-AS1 in NPC samples was significantly higher than that in adjacent normal samples. Moreover, it was predicted that SMAD5-AS1 was mainly expressed in the cytoplasm (Figure 1D), suggesting that SMAD5-AS1 might play a regulatory role through functioning as ceRNA. In order to investigate the downstream mechanism of SMAD5-AS1, prediction was carried out using the RNA22 database and miRcode database. Thirty-six SMAD5-AS1 potential regulatory miRNAs were predicted in the miRcode database, and 1123 miRNAs in the RNA22 database. There were 9 overlapping miRNAs between these two populations of miRNAs (Figure 1E). Expression of these 9 miRNAs in NPC samples was determined by RT-qPCR, which indicated that the expression of miR-195 in NPC samples was remarkably downregulated as compared to the adjacent normal samples with the largest fold change (Figure 1F). Subsequently, the potential target genes of miR-195 were predicted using miRDB and the four other databases. Next, 38 overlapping genes were selected through intersecting the predicted target genes scoring over 75 in the miRDB database, scoring over 0.85 in the mirDIP database and the top 50% in the other databases (Figure 1G). Furthermore, it has been previously reported that the SMAD5 gene plays an important regulatory role in a variety of tumors (15, 16). The KEGG database indicates that SMAD5 is closely associated with the TGF-β signaling pathway (map04350), which is considered to be one of the key pathways in the regulation of NPC progression (17–19). In addition, the expression of SMAD5 in the GSE53819 and GSE64634 datasets was analyzed, indicating that the expression of SMAD5 was much higher in NPC samples than that in adjacent normal samples (Figures 1H,I). Based on those results, we speculated that SMAD5-AS1 might mediate the expression of SMAD5 by regulating miR-195, ultimately affecting the development of NPC.

### SMAD5-AS1 and SMAD5 Are Upregulated but miR-195 Is Downregulated in NPC Tissues and Cells

**Figure 2:**
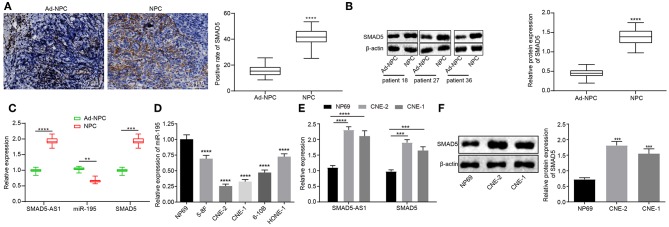
NPC tissues and cells exhibited upregulated SMAD5-AS1 and SMAD5 but downregulated miR-195. **(A)** The positive expression of SMAD5 protein in NPC tissues and adjacent normal tissues detected by immunohistochemistry (400 ×; scale bar = 25 um; *n* = 41). ****p < 0.0001 vs. adjacent normal tissues. **(B)** Protein expression of SMAD5 in NPC tissues and adjacent normal tissues detected by western blot analysis. ****p < 0.0001 vs. adjacent normal tissues. **(C)** The expression of SMAD-AS1, SMAD5, and miR-195 in NPC tissues and adjacent normal tissues determined by RT-qPCR. ****p < 0.0001, ***p < 0.001, and **p < 0.01 vs. adjacent normal tissues. **(D)** The expression of miR-195 in five NPC cell lines and a normal cell line measured using RT-qPCR. ****p < 0.0001 vs. NP69 cells. **(E)** The expression of SMAD5 mRNA and SMAD5-AS1 in CNE-2, CNE-1, and NP69 cells determined by RT-qPCR. ****p < 0.0001, ***p < 0.001 vs. NP69 cells. **(F)** Expression of SMAD5 protein in CNE-2, CNE-1, and NP69 cells determined by western blot analysis. ***p < 0.001 vs. NP69 cells. The data were measurement data, which were presented as mean ± standard deviation. Paired *t*-test was performed for comparisons between adjacent normal tissues and NPC tissues, independent sample *t*-test was applied to compare the differences between two groups, and one-way analysis of variance was employed to compare the differences among multiple groups, followed by Tukey's *post-hoc* test. The experiment was repeated three times.

In order to further confirm the results of microarray-based gene expression profiling, immunohistochemistry, western blot analysis, and RT-qPCR were employed to detect the expression of SMAD5-AS1, SMAD5, and miR-195 in NPC tissues and NPC cell lines. The results displayed that the expression of SMAD5 in NPC tissues was markedly higher than that in adjacent normal tissues (*p* < 0.0001; Figures 2A,B; Supplementary Figure 1). SMAD5-AS1 and SMAD5 were upregulated (*p* < 0.0001, *p* < 0.001) but miR-195 was downregulated (*p* < 0.01) in NPC tissues when compared with adjacent normal tissues (Figure 2C). Chi-square test analysis revealed that upregulated expression of SMAD5 and SMAD5-AS1 and downregulated expression of miR-195 were correlated to larger tumor size, lymph node metastasis and advanced tumor stage (Table 2). The expression of miR-195 was lower in the CNE-2 and CNE-1 cells than that in the NP69 cells (*p* < 0.0001; Figure 2D). Compared with NP69 cells, SMAD5-AS1 and SMAD5 were expressed at high levels in CNE-2 and CNE-1 cells (*p* < 0.0001, *p* < 0.001; Figure 2E). In contrast to NP69 cells, CNE-2 and CNE-1 cells exhibited high protein expression of SMAD5 (*p* < 0.0001; Figure 2F). Based on these results, it could be concluded that SMAD5-AS1 and SMAD5 were highly expressed while miR-195 was poorly expressed in NPC tissues and cells, and CNE-2 and CNE-1 cells were selected for subsequent experiments.

**Table 2:** Associations between the expression of SMAD5, SMAD5-AS1, and miR-195 and the clinicopathological characteristics of patients with NPC.

**Characteristics**	**Case**	**SMAD5 expression**		***p*-value**	**SMAD5-AS1 expression**		***p*-value**	**miR-195 expression**		***p*-value**
		**Low**	**High**	**(Chi-square test)**	**Low**	**High**	**(Chi-square test)**	**Low**	**High**	**(Chi-square test)**
Gender				0.368			0.368			0.658
Male	28	13	15		13	15		15	13	
Female	13	8	5		8	5		6	7	
Age (years)				0.910			0.228			0.440
<44	14	7	7		9	5		6	8	
≥44	27	14	13		12	15		15	12	
T stage				0.001^*^			0.008^*^			<0.001^*^
≤T2	12	11	1		10	2		1	11	
>T2	29	10	19		11	18		20	9	
N stage				0.019^*^			0.028^*^			0.008^*^
N0	4	3	1		4	0		1	3	
N1	12	10	2		8	4		2	10	
N2	12	4	8		6	6		9	3	
N3	13	4	9		3	10		9	4	
Distant metastasis (at diagnosis)				0.002^*^			0.015^*^			0.015^*^
Yes	25	8	17		9	16		18	7	
No	16	13	3		12	4		3	13	
Lymph node metastasis				<0.001^*^			0.015^*^			0.001^*^
Yes	16	16	0		12	4		3	13	
No	25	8	17		9	16		18	7	

* *p < 0.05; SMAD5, drosophila mothers against decapentaplegic protein; SMAD5-AS1, drosophila mothers against decapentaplegic protein antisense RNA 1; miR-195, microRNA-195; NPC, nasopharyngeal carcinoma*.

### SMAD5-AS1 Upregulates SMAD5 Expression by Competitively Binding to miR-195

**Figure 3:**
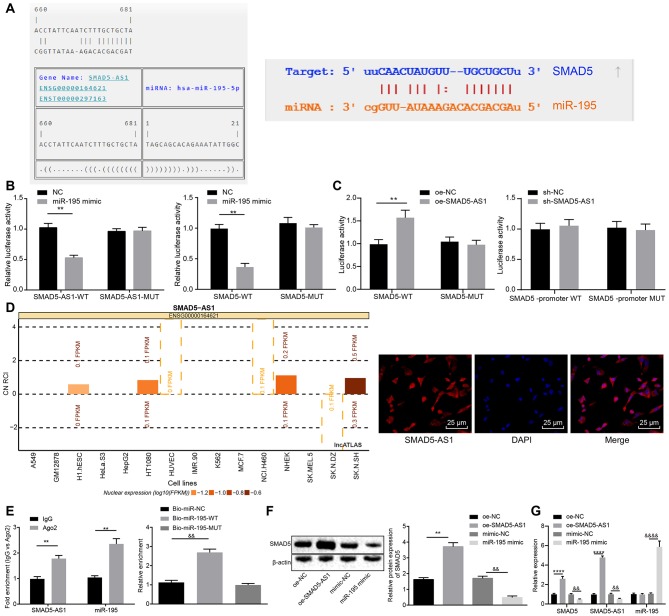
SMAD5-AS1 elevated the expression of SMAD5 by competitively binding to miR-195. **(A)** The relationship between miR-195 and SMAD5-AS1 analyzed using RNA22 website and that between miR-195 and SMAD5 analyzed using starBase website. In **(A)**, the sequence of SMAD5 or SMAD5-AS1 is presented on the top in each pair, and that of miR-195 is at the bottom in each pair. **(B)** The binding of miR-195 to SMAD5-AS1 or SMAD5 in HEK-293T cells verified by dual luciferase reporter gene assay. **p < 0.01 vs. the NC group. **(C)** The relationship between SMAD5-AS1 and SMAD5 in HEK-293T cells analyzed by dual luciferase reporter gene assay. **p < 0.01 vs. the oe-NC group. **(D)** The subcellular localization of SMAD5AS-1 in CNE-2 cells predicted using the website (http://lncatlas.crg.eu/) and FISH assay (400 ×, scale bar = 25 um). **(E)** Binding of SMAD5-AS1 and miR-195 to AGO2 analyzed by RIP assay, and SMAD5-AS1 enrichment in CNE-2 cells detected by RNA-pull down assay. **p < 0.01 vs. the IgG group; ^&&^*p* < 0.01 vs. the Bio-miR-NC group. **(F)** Protein expression of SMAD5 in CNE-2 cells after transfection with oe-SMAD5-AS1 or miR-195 mimic detected by western blot analysis. **p < 0.01 vs. the oe-NC group; ^&&^*p* < 0.01 vs. the mimic-NC group. **(G)** Expression of SMAD5-AS1, SMAD5, and miR-195 in CNE-2 cells transfected with oe-SMAD5-AS1 or miR-195 mimic determined by RT-qPCR. **p < 0.01 vs. the oe-NC group; ^&&^*p* < 0.01 vs. the oe-NC group. ^&*&&&*^*p* < 0.01 vs. the mimic-NC group. The data were measurement data, which were expressed as mean ± standard deviation. Independent sample *t*-test was applied to compare the differences between two groups, and one-way analysis of variance was employed to compare the differences among multiple groups, followed by Tukey's *post-hoc* test. The experiment was repeated three times.

Here, a series of experiments were conducted to analyze the interaction between miR-195 and SMAD5-AS1 or SMAD5. Binding sites were predicted initially using the online predict software RNA22 database (https://cm.jefferson.edu/rna22/Interactive/) and starBase database (http://starbase.sysu.edu.cn/), respectively. The results displayed binding sites between miR-195 and SMAD5-AS1, as well as between miR-195 and SMAD5 (Figure 3A). In addition, according to the results from dual-luciferase reporter gene assay, compared with the NC group, the luciferase activity of SMAD5-AS1-WT plasmid was decreased in HEK-293T cells in the miR-195 mimic group (*p* < 0.05), while the luciferase activity of SMAD5-AS1-MUT plasmid had no obvious changes (*p* > 0.05). The luciferase activity of SMAD5-AS1-WT plasmids showed a significant reduction in the miR-195 mimic group (*p* < 0.05), while the luciferase activity of SMAD5-MUT plasmid indicated no marked changes (*p* > 0.05) (Figure 3B) in comparison to the NC group. These findings suggested a binding relationship between miR-195 and SMAD5-AS1 or SMAD5.

In addition, to confirm whether SMAD5-AS1 directly affected SMAD5, we conducted dual-luciferase reporter gene assay by detecting the luciferase activities of SMAD5-WT and SMAD5-MUT in the presence of SMAD 5-AS1. The luciferase activity of SMAD5-WT plasmid in the SMAD 5-AS1 group was increased when compared with the oe-NC group (*p* < 0.05) and the luciferase activity of SMAD5-MUT plasmid showed no changes (*p* > 0.05). Moreover, neither overexpression of SMAD5-AS1 nor silencing of SMA5-AS1 affected the luciferase activity of the SMAD5 promoter region (Figure 3C). This indicated that SMAD5-AS1 did not direct target SMAD5. Next, the website (http://lncatlas.crg.eu/) was adopted to predict the subcellular localization of SMAD5AS-1. The results indicated that SMAD5-AS1 was mainly located in the cytoplasm of different cell lines, which were consistent with the obtained results of RNA-FISH analysis (Figure 3D). The results of RIP assay showed that SMAD5-AS1 bound by AGO2 was more abundant compared with that bound by IgG (*p* < 0.05), indicating that SMAD5-AS1 could bind to AGO2 protein, that is, miR-195 could bind to SMAD5-AS1 (Figure 3E; Supplementary Figure 2A). Based on RNA pull-down assay, SMAD5-AS1 enrichment had no marked difference in the Bio-miR-195-MUT group compared with the Bio-probe NC group (*p* > 0.05), but SMAD5-AS1 enrichment in the Bio-195-WT group was increased (*p* < 0.01) (Figure 3E; Supplementary Figure 2A), suggesting that SMAD5-AS1 could be enriched around Bio-miR-195-WT, and SMAD5-AS1 could compete for the binding sites of miR-195. After that, western blot analysis was employed to detect the expression of SMAD5 in different groups, and the results showed that compared with the oe-NC group, the expression of SMAD5 in the oe-SMAD5-AS1 group was upregulated (*p* < 0.05); compared with the mimic-NC group, the expression of SMAD5 in the miR-195 mimic group was downregulated (*p* < 0.05) (Figure 3F; Supplementary Figure 2B). Subsequent RT-qPCR results showed that compared with the oe-NC group, the expression of SMAD5-AS1 and SMAD5 in the oe-SMAD5-AS1 group was upregulated (*p* < 0.05). In comparison with the mimic-NC group, the expression of SMAD5-AS1 and SMAD5 was downregulated while the expression of miR-195 was elevated in the miR-195 mimic group (*p* < 0.05) (Figure 3G; Supplementary Figure 2C). It was suggested that miR-195 could inhibit the expression of SMAD5-AS1 and SMAD5, while SMAD5-AS1 and SMAD5 had no effects on the expression of miR-195. These results together suggested that SMAD5-AS1 could competitively bind to miR-195 to upregulate the expression of SMAD5.

### Depleted SMAD5-AS1 or SMAD5 or Overexpressed miR-195 Inhibits NPC Cell Proliferation and Promotes Apoptosis

**Figure 4:**
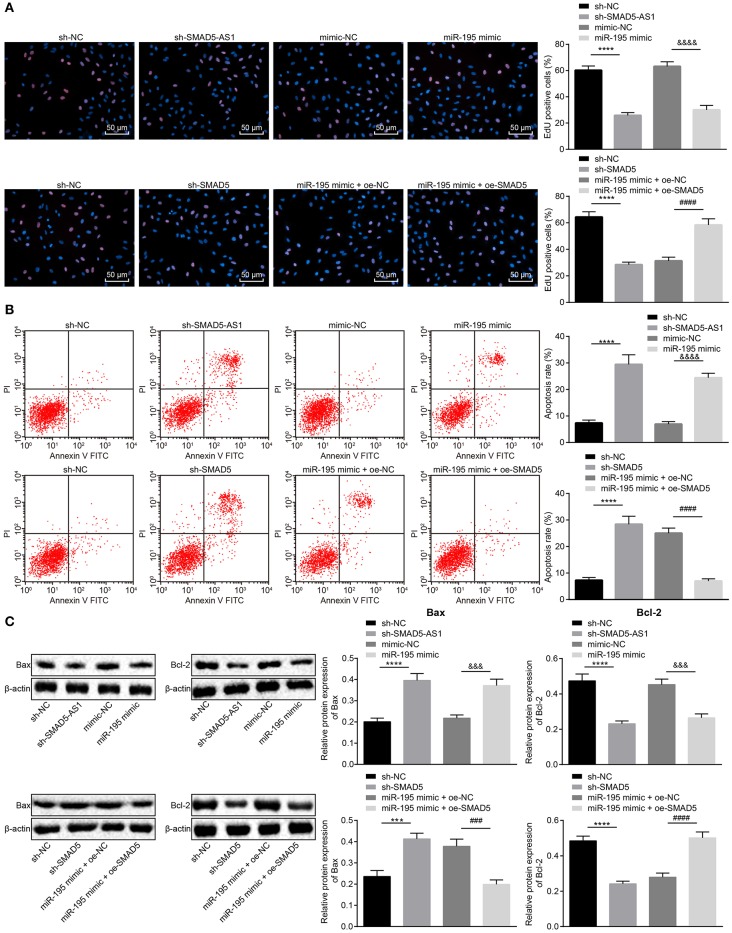
CNE-2 cell proliferation was suppressed while cell apoptosis was enhanced following knockdown of SMAD5-AS1 or SMAD5 or elevation of miR-195. **(A)** CNE-2 cell proliferation assessed by EdU assay (200 ×, scale bar = 50 um). **(B)** CNE-2 cell apoptosis rate measured using flow cytometry. In **(A,B)**, ****p < 0.0001 vs. the sh-NC group; ^&*&&&*^*p* < 0.0001 vs. the mimic-NC group; ^*####*^*p* < 0.0001 vs. the miR-195 mimic + oe-NC group. **(C)** The protein expression of apoptosis-related factors Bax and Bcl-2 in CNE-2 cells determined by western blot analysis. ****p < 0.0001 vs. the sh-NC group; ***p < 0.001 vs. the sh-NC group; ^&*&&*^*p* < 0.0001 vs. the mimic-NC group; ^*####*^*p* < 0.0001 vs. the miR-195 mimic + oe-NC group; ^*###*^*p* < 0.001 vs. the miR-195 mimic + oe-NC group. The data were measurement data and depicted as mean ± standard deviation. Data between two groups were tested using independent sample *t*-test. The experiment was repeated 3 times.

To detect the effects of SMAD5-AS1, miR-195, and SMAD5 on NPC cell proliferation and apoptosis, EdU assay and flow cytometry were adopted to detect the proliferation and apoptosis of CNE-2 and CNE-1 cells, respectively, and western blot analysis was used to detect the protein expression of apoptosis-related factors (Bax and Bcl-2) in CNE-2 and CNE-1 cells. As determined by EdU assay (Figure 4A; Supplementary Figure 3A), compared with the sh-NC group, proliferative CNE-2 and CNE-1 cells in the sh-SMAD5-AS1 group were greatly decreased (*p* < 0.01). The CNE-2 and CNE-1 cell proliferation in the miR-195 mimic group was markedly reduced relative to the mimic NC group (*p* < 0.01). In addition, in comparison with the sh-NC group, the sh-SMAD5 group showed declines in proliferative CNE-2 and CNE-1 cells (*p* < 0.01). However, CNE-2 and CNE-1 cell proliferation was promoted in the miR-195 mimic + oe-SMAD5 group when compared with the miR-195 mimic + oe-NC group (*p* < 0.01).

Results of flow cytometry (Figure 4B; Supplementary Figure 3B) revealed elevated CNE-2 and CNE-1 cell apoptosis rates in the sh-SMAD5-AS1 group in comparison with that in the sh-NC group (*p* < 0.01), while marked increases in CNE-2 and CNE-1 cell apoptosis rates were observed in the miR-195 mimic group in comparison with the mimic NC group (*p* < 0.01). However, compared with the sh-NC group, increases in CNE-2 and CNE-1 cell apoptosis rates were observed in the sh-SMAD5 group (*p* < 0.01). The CNE-2 and CNE-1 cell apoptosis rates in the miR-195 mimic + oe-SMAD5 group were lower than that in the miR-195 mimic + oe-NC group (*p* < 0.01).

Based on the results of western blot analysis (Figure 4C; Supplementary Figure 3C), Bax expression was upregulated while Bcl-2 expression was downregulated in the sh-SMAD5-AS1 group when compared with the sh-NC group (*p* < 0.01, *p* < 0.05). The miR-195 mimic group exhibited elevated Bax expression but reduced Bcl-2 expression vs. the mimic NC group (*p* < 0.05, *p* < 0.01). Additionally, in contrast to the sh-NC group, Bax expression was increased but Bcl-2 expression was decreased in the sh-SMAD5 group (*p* < 0.05, *p* < 0.01). In comparison with the miR-195 mimic + oe-NC group, Bax expression was downregulated while Bcl-2 expression was upregulated in the miR-195 mimic + oe-SMAD5 group (*p* < 0.01). These data demonstrated that silencing of SMAD5-AS1 or SMAD5 or overexpression of miR-195 could inhibit the proliferation and enhance apoptosis of NPC cells.

### Silencing of SMAD5-AS1, Silencing of SMAD5 or Elevation of miR-195 Inhibits NPC Cell Invasion and Migration as Well as EMT

**Figure 5:**
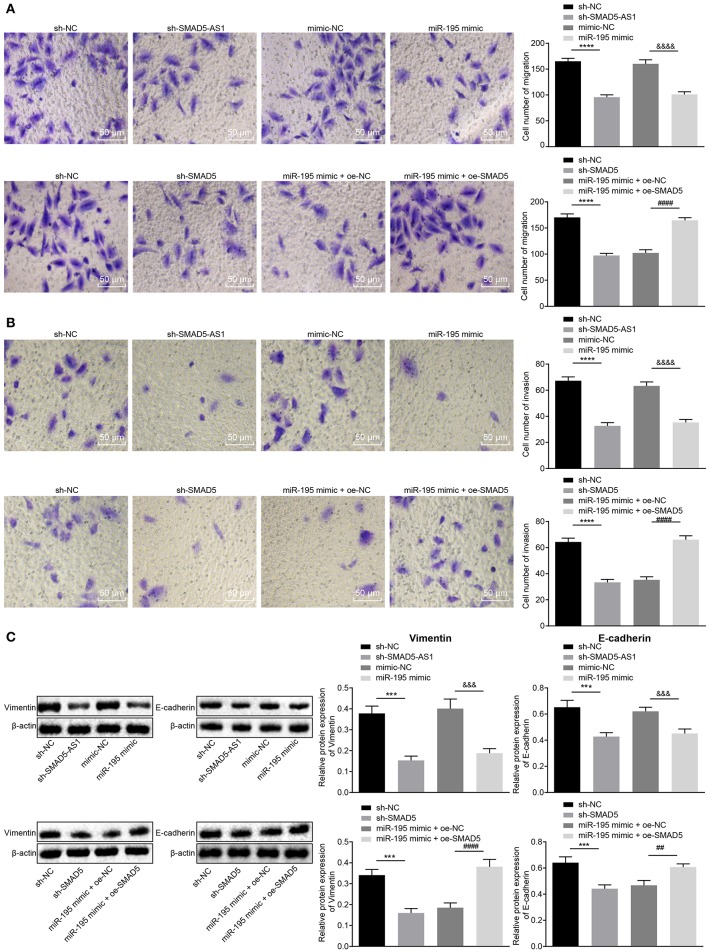
CNE-2 cell invasion, migration, and EMT were inhibited following silencing of SMAD5-AS1 or SMAD5 or upregulation of miR-195. **(A)** CNE-2 cell migration based on Transwell assay (200 ×, scale bar = 50 um). **(B)** CNE-2 cell invasion based on Transwell detection (200 ×, scale bar = 50 um). In **(A,B)**, ****p < 0.0001 vs. the sh-NC group; ^&*&&&*^*p* < 0.0001 vs. the mimic-NC group; ^*####*^*p* < 0.0001 vs. the miR-195 mimic + oe-NC group. **(C)** The protein expression of Vimentin and E-cadherin in CNE-2 cells determined by western blot analysis, ***p < 0.001 vs. the sh-NC group; ^&*&&*^*p* < 0.001 vs. the mimic-NC group; ^*####*^*p* < 0.0001 vs. the miR-195 mimic + oe-NC group; ^*##*^*p* < 0.01 vs. the miR-195 mimic + oe-NC group. The statistical data were measurement data and expressed as mean ± standard deviation. *n* = 6. Independent sample *t*-test was adopted to compare data between two groups. The experiment was repeated 3 times.

To figure out the effect of SMAD5-AS1, miR-195 and SMAD5 on migration, invasion and EMT in NPC cells, Transwell assay was applied to detect CNE-2 and CNE-1 cell migration and invasion abilities, and western blot analysis was adopted to detect the expression of EMT markers Vimentin and E-cadherin in CNE-2 and CNE-1 cells. The results displayed (Figures 5A,B; Supplementary Figures 4A,B) that compared with the sh-NC group, reductions were notable in cell migration and invasion abilities in the sh-SMAD5-AS1 group (*p* < 0.01, *p* < 0.05), and the miR-195 mimic group presented decreased cell migration and invasion abilities vs. the mimic NC group (*p* < 0.05). Furthermore, compared with the sh-NC group, cell migration and invasion abilities were reduced in the sh-SMAD5 group (*p* < 0.05), while increases in cell migration and invasion abilities were found in the miR-195 mimic + oe-SMAD5 group vs. the miR-195 mimic + oe-NC group (*p* < 0.01). As shown in Figure 5C; Supplementary Figure 4C, the expression of Vimentin in CNE-2 and CNE-1 cells was lower while that of E-cadherin was higher in the sh-SMAD5-AS1 group than in the sh-NC group (*p* < 0.01, *p* < 0.05). Compared with the mimic NC group, the miR-195 mimic group displayed reduced expression of Vimentin (*p* < 0.05) but elevated E-cadherin expression (*p* < 0.01). Additionally, compared with the sh-NC group, Vimentin expression was downregulated (*p* < 0.05) while E-cadherin expression was upregulated in the sh-SMAD5 group (*p* < 0.01); the miR-195 mimic + oe-SMAD5 group exhibited elevation in Vimentin expression (*p* < 0.05) but decline in E-cadherin expression relative to the miR-195 mimic + oe-NC group (*p* < 0.01). These findings provided evidence that SMAD5-AS1 silencing or SMAD5 silencing or miR-195 overexpression could repress invasion, migration and EMT in NPC cells.

### Silencing of SMAD5-AS1 or Elevation miR-195 Impairs NPC Cell Growth and Migration via Inhibition of BMP2/SMAD5

**Figure 6:**
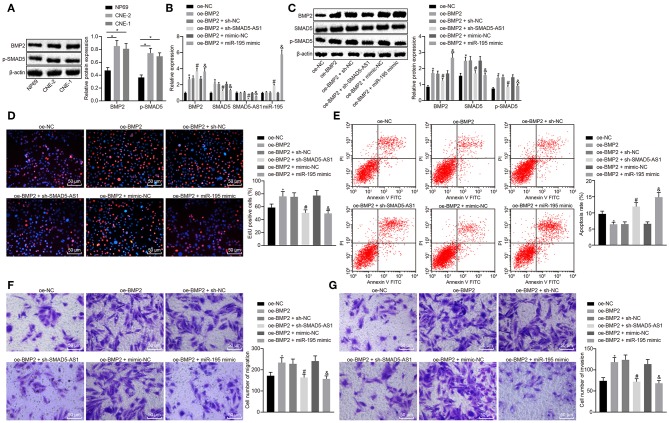
Silencing of SMAD5-AS1 or miR-195 overexpression impeded CNE-2 cell proliferating, migrating and invading abilities *via* blocking the BMP2/SMAD5 pathway. **(A)** The protein expression of BMP2 and phosphorylated SMAD5 in CNE-2 cells measured using western blot analysis. ***p < 0.001 vs. NP69; **p < 0.01 vs. NP69. **(B)** The expression of BMP2, miR-195, SMAD5, and SMAD5-AS1 in CNE-2 cells determined using RT-qPCR. **(C)** The protein expression of BMP2, SMAD5 and phosphorylated SMAD5 in CNE-2 cells measured using western blot analysis. **(D)** CNE-2 cell proliferation assessed by EdU assay. **(E)** CNE-2 cell apoptosis rate measured by flow cytometry. **(F)** CNE-2 cell migration evaluated by Transwell assay (200 ×, scale bar = 50 um). **(G)** CNE-2 cell invasion evaluated by Transwell assay (200 ×, scale bar = 50 um). In **(B–G)**, * vs. the oe-NC group, ^#^*p* < 0.05 vs. the oe-BMP2 + sh-NC group, ^&^*p* < 0.05 vs. the oe-BMP2 + mimic-NC group. The measurement data were expressed as mean ± standard deviation. *n* = 6. Independent sample *t*-test was adopted to compare data between two groups. The experiment was repeated 3 times.

Bone morphogenetic protein 2 (BMP2), as a stimulator of the SMAD5 pathway (20), is reported to accelerate NPC cell migration and invasion as well as to induce EMT (21, 22). We subsequently intended to explore whether the role of SMAD5-AS1/miR-195 in NPC was related to the BMP2/SMAD5 pathway. First of all, the expression of BMP2 protein and phosphorylated SMAD5 in CNE-2 and CNE-1 cell lines was measured using western blot analysis. The results showed that compared with NP69 cells, the expression of BMP2 and phosphorylated SMAD5 was increased in CNE-2 and CNE-1 cells (Figure 6A). Next, CNE-2 and CNE-1 cells were infected with lentivirus expressing oe-BMP2 to overexpress BMP2 and RT-qPCR and Western blot analysis were used to determine the expression of SMAD5-AS1 and miR-195 and mRNA and protein expression of BMP2 and SMAD5 following infection. The results showed that the mRNA and protein expression of BMP2 and SMAD5 in the oe-BMP2 group was much higher than that in the oe-NC group. In addition, the expression of BMP2 and miR-195 was increased while that of SMAD5 and SMAD5-AS1 was lowered in the oe-BMP2 + sh-SMAD5-AS1 group in comparison to the oe-BMP2 + sh-NC group. In comparison to the oe-BMP2 + mimic-NC group, increase in expression of BMP2 and miR-195 as well as reduction in expression of SMAD5 and SMAD5-AS1 was observed in the oe-BMP2 + miR-195 mimic group (Figure 6B; Supplementary Figure 5A). Western blot analysis also showed that knockdown of SMAD5-AS1 or miR-195 overexpression could inhibit the phosphorylation of SMAD5 induced by overexpressed BMP2 (Figure 6C; Supplementary Figure 5B). These results suggested that silencing SMAD5-AS1 or overexpression of miR-195 could inhibit BMP2-induced activation of the SMAD5 signaling pathway. The data obtained from cellular function experiments revealed that compared with the oe-NC group, cell proliferation, migration and invasion were increased, while cell apoptosis was decreased in the oe-BMP2 group. Compared with the oe-BMP2 + sh-NC group or the oe-BMP2 + mimic-NC group, the oe-BMP2 + sh-SMAD5-AS1 group or the oe-BMP2 + miR-195 mimic group showed significant reductions in cell proliferation, migration and invasion capacities but a significant increase in cell apoptosis (Figures 6D–G; Supplementary Figures 5C–F). These results suggested that silencing of SMAD5-AS1 or overexpression of miR-195 could inhibit BMP2-induced cell growth and migration.

### Silencing of SMAD5-AS1 or SMAD5 or Elevation of miR-195 Represses EMT and Tumorigenicity *in vivo*

**Figure 7:**
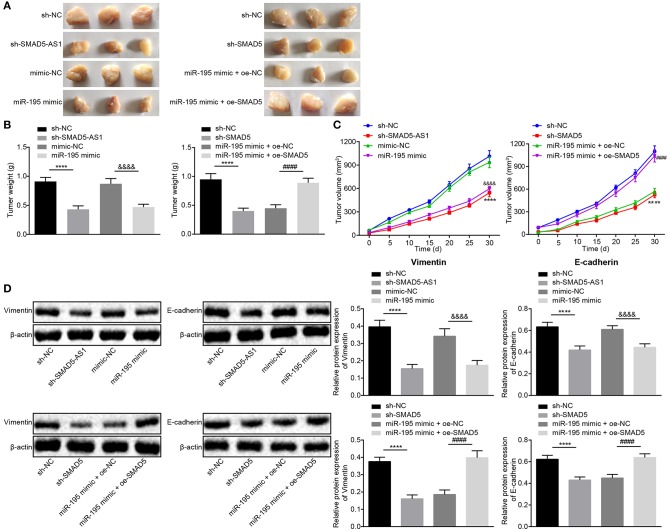
The tumorigenesis and EMT were suppressed in response to depletion of SMAD5-AS1 or SMAD5 or elevation of miR-195 *in vivo*. **(A–C)** Xenograft tumors **(A)** and quantitative analysis of tumor weight **(B)** and volume **(C)** in mice injected with CNE-2 cells that had been infected with lentivirus carrying miR-195 mimic, sh-SMAD5-AS1, and sh-SMAD5. **(D)** Protein expression of EMT-related Vimentin and E-cadherin in mice injected with the infected CNE-2 cells detected by western blot analysis. ****p < 0.0001 vs. the sh-NC group; ^&*&&&*^*p* < 0.0001 vs. the mimic-NC group; ^*####*^*p* < 0.0001 vs. the miR-195 mimic + oe-NC group. The measurement data were expressed as mean ± standard deviation. *n* = 6. Independent sample *t*-test was adopted to compare data between two groups, and repeated measures ANOVA was used to compare the two groups at different time points, followed by Bonferroni post-test. The experiment was repeated 3 times.

The formation of xenograft tumor in nude mice was assessed to detect the effects of SMAD5-AS, SMAD5, and miR-195 on tumor growth. After CNE-2 cell suspensions were subcutaneously injected into the dorsal surface at the crotch of right hind limb in nude mice, their eating, activity and mental status were observed every week. After 30 days, the tumor tissues were excised from the nude mice after euthanasia (Figure 7A). Following the measurement of the weight of tumors, the results displayed (Figure 7B) that compared with the sh-NC group, there was an evident decrease in tumor weight in the sh-SMAD5-AS1 group (*p* < 0.05), and the tumor weight was lower in the miR-195 mimic group than in the mimic NC group (*p* < 0.05). In addition, compared with the sh-NC group, the findings showed decreased tumor weight in the sh-SMAD5 group (*p* < 0.05), while there was elevated tumor weight in the miR-195 mimic + oe-SMAD5 group relative to the miR-195 mimic + oe-NC group (*p* < 0.05).

According to the tumor volume changes in nude mice measured every 5 days, the line graph was drawn as Figure 7C. The sh-SMAD5-AS1 group showed reduced tumor volume vs. the sh-NC group (*p* < 0.05), which was in line with the reduction shown in the miR-195 mimic group and the sh-SMAD5 group when compared with the mimic NC group and the sh-NC group, respectively (all *p* < 0.05). However, in contrast to the miR-195 mimic + oe-NC group, tumor volume was increased in the miR-195 mimic + oe-SMAD5 group (*p* < 0.05).

Based on the results of EMT-related Vimentin and E-cadherin protein expression measured by western blot analysis, protein expression of Vimentin was downregulated (*p* < 0.05), while that of E-cadherin was upregulated in the sh-SMAD5-AS1 group vs. the sh-NC group (*p* < 0.01). In comparison with the mimic NC group, the miR-195 mimic group exhibited a decline in protein expression of Vimentin (*p* < 0.01) but elevation in the protein expression of E-cadherin (*p* < 0.05). Besides, compared with the sh-NC group, decreased Vimentin but increased E-cadherin were displayed in the sh-SMAD5 group (*p* < 0.05). The expression of Vimentin was higher (*p* < 0.01) while expression of E-cadherin was lower (*p* < 0.05) in the miR-195 mimic + oe-SMAD5 group than in the miR-195 mimic + oe-NC (Figure 7D). Taken together, SMAD5-AS1 or SMAD5 depletion and miR-195 overexpression could inhibit tumorigenesis and EMT in nude mice.

## Discussion

NPC is a rare malignancy with the characteristic of specific geographic spread and presents a high incidence in southern China (23). A prior study has noted disordered lncRNA expression in various human cancers, including NPC, lung and breast cancers, which also verified lncRNA AFAP1-AS1 had a strong relationship with NPC progression and poor prognosis of NPC (24). However, in reviewing the literature, little data were found on the association between SMAD5-AS1 and NPC. The present study focused on the effect of SMAD5-AS1 on NPC.

Our initial results showed that NPC tissues and cells exhibited high levels of SMAD5-AS1 and SMAD5 but low levels of miR-195 as compared to normal controls. In line with our findings, it has been reported that aberrant expression of lncRNAs is associated with the development of NPC (25). For instance, lncRNA-ROR has a strong association with the proliferation, metastasis and apoptosis of NPC cells (6). Although the dysregulation of SMAD5-AS1 has not been mentioned in NPC yet, its aberrant expression in human non-small-cell lung carcinoma (NSCLC) has recently been identified (26). Recently, miRNAs have been reported to mediate various biological processes and exert great effects on cancer development (27). miR-195 is found to be downregulated in bladder cancer relative to corresponding normal urothelium (11). In agreement with our findings, miR-195 is observed to be decreased in NPC tissues (28). SMAD5 has been implicated as a downstream signal mediator for bone morphogenetic proteins (BMPs) (29). Expression of phosphorylated SMAD3, another member of the SMAD signaling pathway, is found to be inhibited in NPC CNE-2 cells (30). Inhibition of SMAD3 is reported to be involved in repression of EMT progress induced by miR-92b in NPC cells (31). Similarly, our results indicated that SMAD5-AS1 was found to regulate SMAD5 by competitively binding to miR-195. LncRNAs serve as competing endogenous RNAs (ceRNAs) of miRNAs and then participate in the mediation of protein coding gene expression in some cancers (32). The expression of miR-195 could be reduced by overexpression of lncRNA PVT1, while silencing of PVT1 could upregulate miR-195 expression (33). Previous evidence has revealed that Raf-1 is a new direct target of miR-195, and the expression of Raf-1 is markedly decreased by miR-195 (34). Similarly, SMAD5-AS1 is reported to be a ceRNA of miR-135b-5p to upregulate APC expression in diffuse large B cell lymphoma (35). Subsequently, the findings of this study showed that SMAD5-AS1 upregulated SMAD5 *via* competitively binding to miR-195 in NPC.

Furthermore, our results demonstrated that depletion of SMAD5-AS1 or SMAD5 or overexpression of miR-195 inhibited the proliferation, invasion, migration, EMT and promoted apoptosis in NPC cells, evidenced by decreased protein expression of Vimentin and Bcl-2 (anti-apoptotic genes) but elevated expression of Bax (pro-apoptotic genes) (36). The complicated process of EMT is associated with the loss of epithelial marker E-cadherin and acquisition of mesenchymal marker Vimentin (37). A previous study has demonstrated that activation of the SMAD5 signaling pathway could promote proliferation of endothelial cells (38). In addition, silencing of SMAD5 could enhance apoptosis of human granulosa cells *via* the FasL-Fas signaling pathway (39). Moreover, the inhibited phosphorylation of SMAD3 can induce cell cycle arrest and cell apoptosis in NPC (30). Furthermore, overexpression of miR-195 exerts an inhibitory effect on proliferation, migration and invasion of NSCLC cells (40). MiR-195 could also promote apoptosis in mouse podocytes treated with high glucose by inhibiting Bcl-2 (41). Furthermore, our *in vivo* results indicated that silencing of SMAD5-AS1 or SMAD5 or elevation of miR-195 could repress tumor growth. Similarly, high expression of miR-195 suppresses tumor growth in prostate carcinoma *in vivo* (42). SMAD5 could serve as a target of miRNA to inhibit the angiogenesis, thereby contributing to tumor growth and metastases (43).

Moreover, our study further demonstrated the effects of SMAD5-AS1 and miR-195 on the BMP2/SMAD5 pathway. The translocation of pSMAD5 from the cytoplasm to the nucleus can be stimulated by BMP2 (20). Consistently, BMP2 overexpression was determined to stimulate the phosphorylation of SMAD5 in this study. Grunhagen et al. have identified that miR-195-5p impairs the induction of BMP responsive genes and blocks the BMP/SMAD-pathway in a dose-dependent manner (44). It was proven in the present study that silencing of SMAD5-AS1 or overexpression of miR-195 could inhibit BMP2-induced cell growth and migration, suggesting a regulatory network of SMAD5-AS1/miR-195/BMP2/SMAD5. However, this briefly discussed finding remains to be explored in the future.

**Figure 8:**
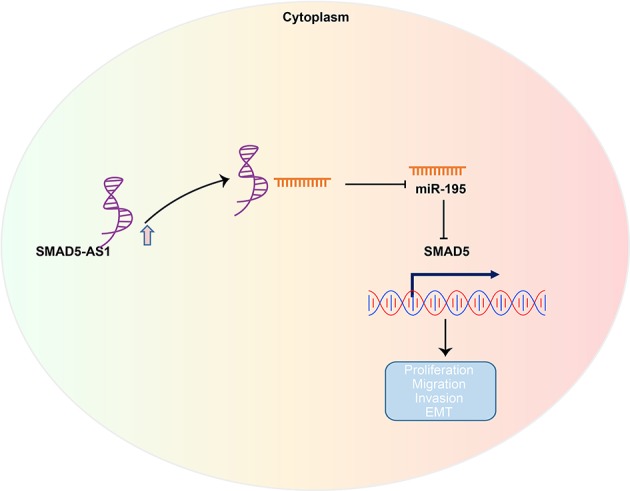
The regulatory network and function of SMAD5-AS1 in NPC. LncRNA SMAD5-AS1 upregulates SMAD5 expression by competitively binding to miR-195, thereby promoting EMT of NPC.

In summary, our results suggested that silencing of SMAD5-AS1 exerts an inhibitory effect on EMT of NPC cells by downregulating SMAD5 *via* overexpression of miR-195 (Figure 8). These results facilitate enhanced understanding of the association of SMAD5-AS1, miR-195, and SMAD5 in NPC cells and provide a potential target for the treatment of NPC. Nevertheless, further studies are required due to the lack of clinical experiments on the regulatory role of SMAD5-AS1, SMAD5, and miR-195.

## Data Availability Statement

The raw data supporting the conclusions of this manuscript will be made available by the authors, without undue reservation, to any qualified researcher.

## Ethics Statement

This study was approved by the Ethics Committee of Tongji Huangzhou Hospital, Huazhong University of Science and Technology. Informed written consent was obtained from each patient prior to the study. Animal experiments were conducted in strict accordance with the Guide for the Care and Use of Laboratory animals published by the US National Institutes of Health (NIH). Great efforts were made to minimize the number of animals used in the experiments and their suffering.

## Author Contributions

SL, BZ, HZ, CS, MZ, JP, BK, and GD designed the study. SL, BZ, HZ, CS, and XX collated the data, carried out data analyses, and produced the initial draft of the manuscript. MZ, JP, BK, and GD contributed to drafting the manuscript. All authors have read and approved the final submitted manuscript.

### Conflict of Interest

The authors declare that the research was conducted in the absence of any commercial or financial relationships that could be construed as a potential conflict of interest.

## Supplementary Material

Supplementary Figure 1 Western blot analysis of SMAD5 protein in NPC and adjacent normal tissues (*n* = 38).

Supplementary Figure 2 Relationship between miR-195, SMAD5-AS1, and SMAD5 in CNE-1 cells. **(A)** Binding of SMAD5-AS1 and miR-195 to AGO2 by RIP assay, and SMAD5-AS1 enrichment in CNE-1 cells detected by RNA-pull down assay. ^**^*p* < 0.01 vs. the IgG group; ^*^*p* < 0.05 vs. the IgG group; ^&*&&*^*p* < 0.001 vs. the Bio-miR-NC group. **(B)** Protein expression of SMAD5 in CNE-1 cells transfected with oe-SMAD5-AS1 or miR-195 mimic measured by western blot analysis. ^****^*p* < 0.0001 vs. the sh-NC group; ^&*&&*^*p* < 0.001 vs. the mimic-NC group. **(C)** Expression of SMAD5-AS1, SMAD5, and miR-195 in CNE-1 cells transfected with oe-SMAD5-AS1 or miR-195 mimic determined by RT-qPCR. ^****^*p* < 0.0001 vs. the sh-NC group; ^**^*p* < 0.01 vs. the sh-NC group; ^&*&&&*^*p* < 0.0001 vs. the mimic-NC group; ^&*&&*^*p* < 0.001 vs. the mimic-NC group; ^&&^*p* < 0.01 vs. the mimic-NC group. The measurement data were expressed as mean ± standard deviation. *n* = 6. Independent sample *t*-test was adopted to compare data between two groups, and one-way ANOVA was used to compare the data among multiple groups, followed by Tukey's *post-hoc* tests with corrections for multiple comparisons. The experiment was repeated 3 times.

Supplementary Figure 3 SMAD5-AS1 and SMAD5 silencing or miR-195 overexpression impeded CNE-1 cell proliferation and enhances cell apoptosis. **(A)** CNE-1 cell proliferation assessed by EdU assay (200 ×, scale bar = 50 um). ^***^*p* < 0.001 vs. the sh-NC group; ^**^*p* < 0.01 vs. the sh-NC group; ^&*&&*^*p* < 0.001 vs. the mimic-NC group; ^*##*^*p* < 0.01 vs. the miR-195 mimic + oe-NC group. **(B)** CNE-1 cell apoptosis rate measured using flow cytometry. ^****^*p* < 0.0001 vs. the sh-NC group; ^&*&&*^*p* < 0.001 vs. the mimic-NC group; ^*####*^*p* < 0.0001 vs. the miR-195 mimic + oe-NC group. **(C)** The protein expression of apoptosis-related factors Bax and Bcl-2 in CNE-1 cells determined by western blot analysis. ^***^*p* < 0.001 vs. the sh-NC group; ^**^*p* < 0.01 vs. the sh-NC group; ^&^*p* < 0.05 vs. the mimic-NC group; ^*###*^*p* < 0.001 vs. the miR-195 mimic + oe-NC group; ^*##*^*p* < 0.01 vs. the miR-195 mimic + oe-NC group. The measurement data were depicted as mean ± standard deviation. Data between two groups were tested using independent sample *t*-test. The experiment was repeated 3 times.

Supplementary Figure 4 SMAD5-AS1 and SMAD5 silencing or miR-195 overexpression disrupted CNE-1 cell invasion, migration and reversed EMT. **(A)** CNE-1 cell migration measured by Transwell assay (200 ×, scale bar = 50 um). ^****^*p* < 0.0001 vs. the sh-NC group; ^&*&&&*^*p* < 0.0001 vs. the mimic-NC group; ^*####*^*p* < 0.0001 vs. the miR-195 mimic + oe-NC group. **(B)** CNE-1 cell invasion measured by Transwell assay (200 ×, scale bar = 50 um). ^****^*p* < 0.0001 vs. the sh-NC group; ^**^*p* < 0.01 vs. the sh-NC group; ^&&^*p* < 0.01 vs. the mimic-NC group; ^*##*^*p* < 0.01 vs. the miR-195 mimic + oe-NC group. **(C)** The protein expression of Vimentin and E-cadherin in CNE-1 cells determined by western blot analysis, ^**^*p* < 0.01 vs. the sh-NC group; ^*^*p* < 0.05 vs. the sh-NC group; ^&&^*p* < 0.01 vs. the mimic-NC group; ^*##*^*p* < 0.01 vs. the miR-195 mimic + oe-NC group; ^#^*p* < 0.05 vs. the miR-195 mimic + oe-NC group. The measurement data were expressed as mean ± standard deviation. *n* = 6. Independent sample *t*-test was adopted to compare data between two groups. The experiment was repeated 3 times.

Supplementary Figure 5 Silencing of SMAD5-AS1 or miR-195 overexpression impeded CNE-1 cell growth and migration *via* blocking the BMP2/SMAD5 pathway. **(A)** The expression of BMP2, miR-195, SMAD5, and SMAD5-AS1 in CNE-1 cells determined using RT-qPCR. **(B)** The protein expression of BMP2 and phosphorylated SMAD5 in CNE-1 cells measured using western blot analysis. **(C)** CNE-1 cell proliferation assessed by EdU assay. **(D)** CNE-1 cell apoptosis rate measured by flow cytometry. **(E)** CNE-1 cell migration measured by Transwell assay (200 ×, scale bar = 50 um). **(F)** CNE-1 cell invasion measured by Transwell assay (200 ×, scale bar = 50 um). ^*^ vs. the oe-NC group, ^#^*p* < 0.05 vs. the oe-BMP2 + sh-NC group, ^&^*p* < 0.05 vs. the oe-BMP2 + mimic-NC group. The measurement data were expressed as mean ± standard deviation. *n* = 6. Independent sample *t*-test was adopted to compare data between two groups. The experiment was repeated 3 times.
