# Supplementary figures and images for: Silencing of Long Non-coding RNA SMAD5-AS1 Reverses Epithelial Mesenchymal Transition in Nasopharyngeal Carcinoma via microRNA-195-Dependent Inhibition of SMAD5
Source: Front Oncol. 2019 Dec 13;9:1246. doi: 10.3389/fonc.2019.01246 (PMC6923203; doi:10.3389/fonc.2019.01246)

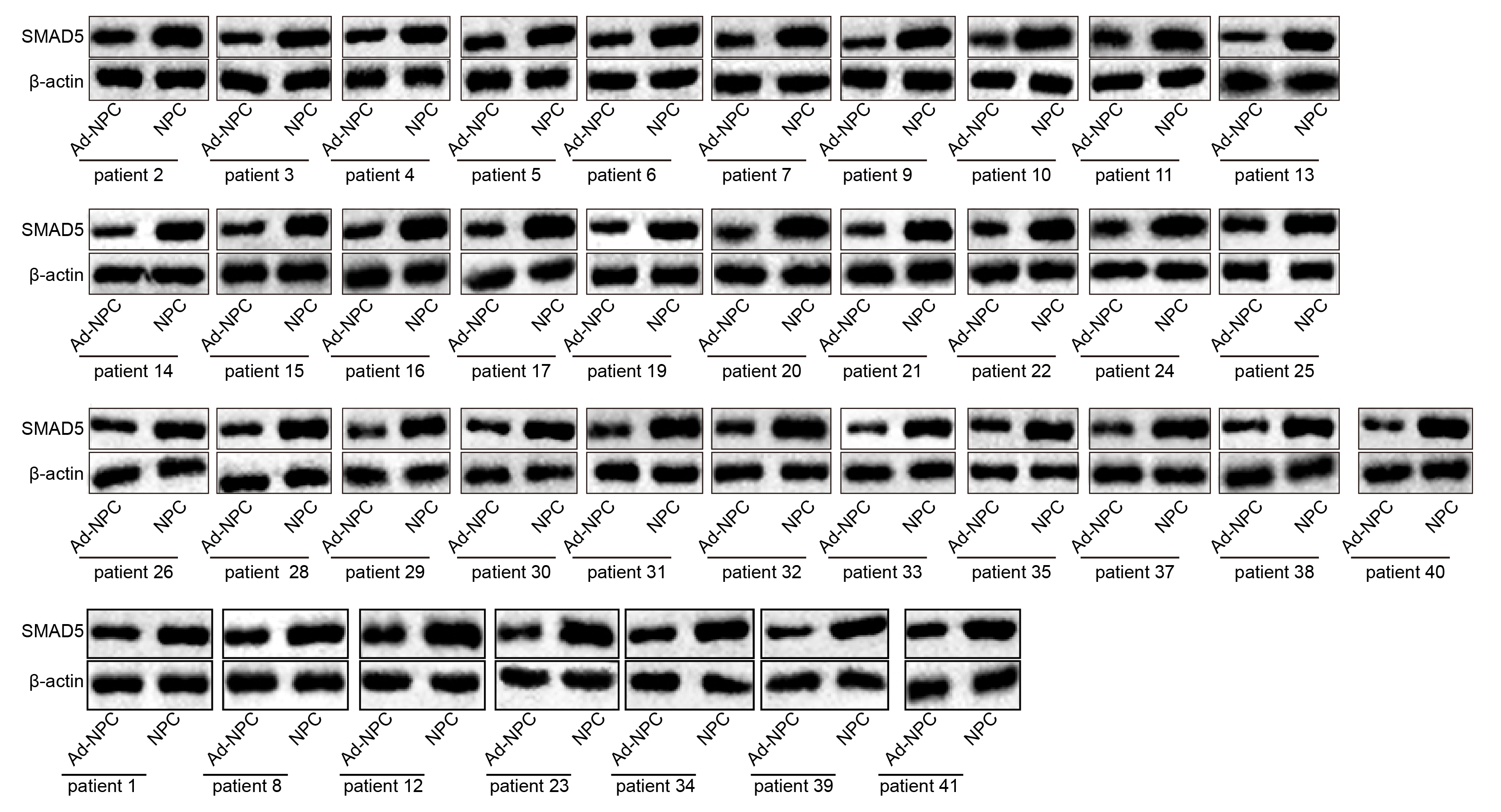

Supplement: Supplementary Figure 1 — Western blot analysis of SMAD5 protein in NPC and adjacent normal tissues (n = 38). [file Image_1.JPEG]

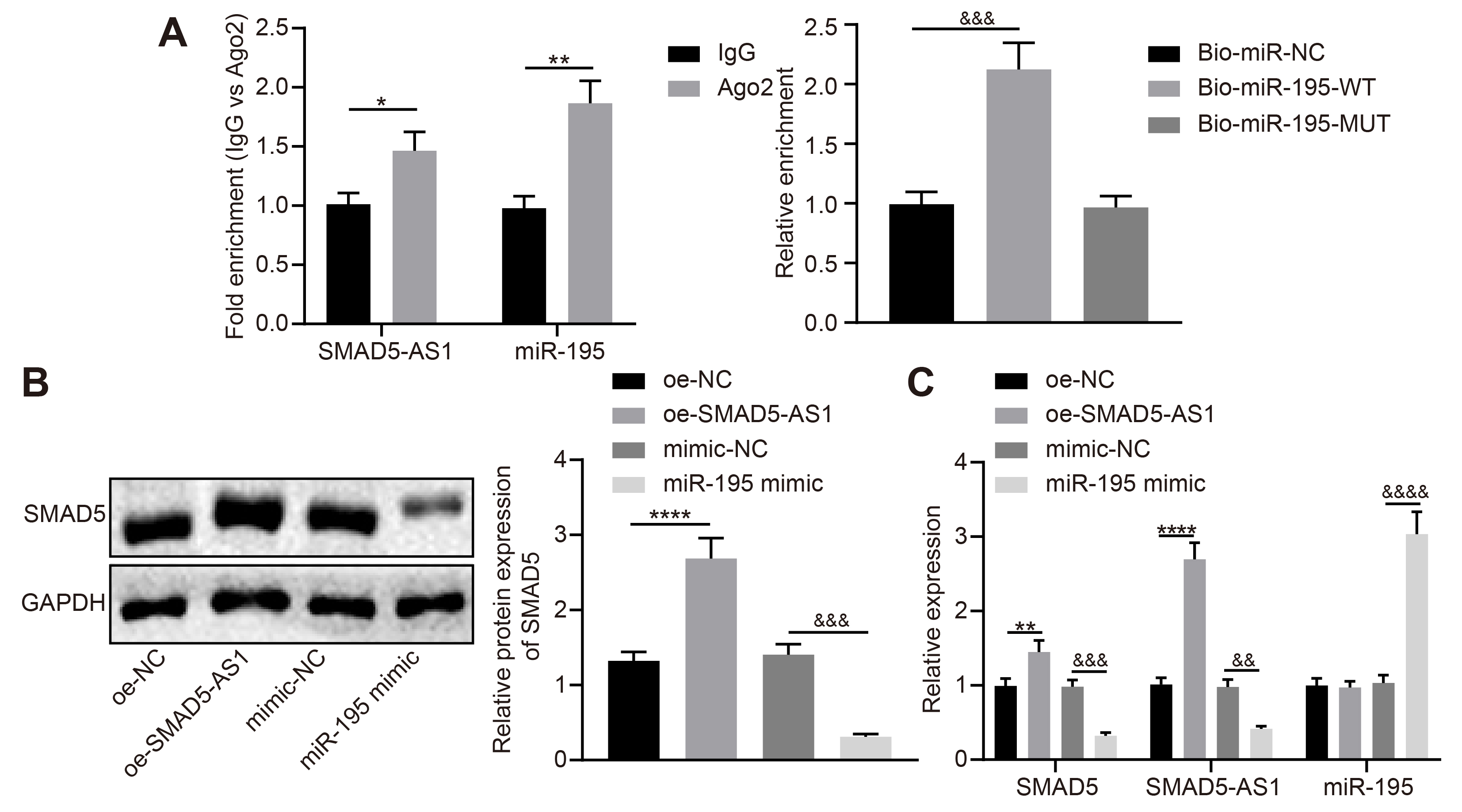

Supplement: Supplementary Figure 2 — Relationship between miR-195, SMAD5-AS1, and SMAD5 in CNE-1 cells. (A) Binding of SMAD5-AS1 and miR-195 to AGO2 by RIP assay, and SMAD5-AS1 enrichment in CNE-1 cells detected by RNA-pull down assay. **p < 0.01 vs. the IgG group; *p < 0.05 vs. the IgG group; &&&p < 0.001 vs. the Bio-miR-NC group. (B) Protein expression of SMAD5 in CNE-1 cells transfected with oe-SMAD5-AS1 or miR-195 mimic measured by western blot analysis. ****p < 0.0001 vs. the sh-NC group; &&&p < 0.001 vs. the mimic-NC group. (C) Expression of SMAD5-AS1, SMAD5, and miR-195 in CNE-1 cells transfected with oe-SMAD5-AS1 or miR-195 mimic determined by RT-qPCR. ****p < 0.0001 vs. the sh-NC group; **p < 0.01 vs. the sh-NC group; &&&&p < 0.0001 vs. the mimic-NC group; &&&p < 0.001 vs. the mimic-NC group; &&p < 0.01 vs. the mimic-NC group. The measurement data were expressed as mean ± standard deviation. n = 6. Independent sample t-test was adopted to compare data between two groups, and one-way ANOVA was used to compare the data among multiple groups, followed by Tukey's post-hoc tests with corrections for multiple comparisons. The experiment was repeated 3 times. [file Image_2.jpg]

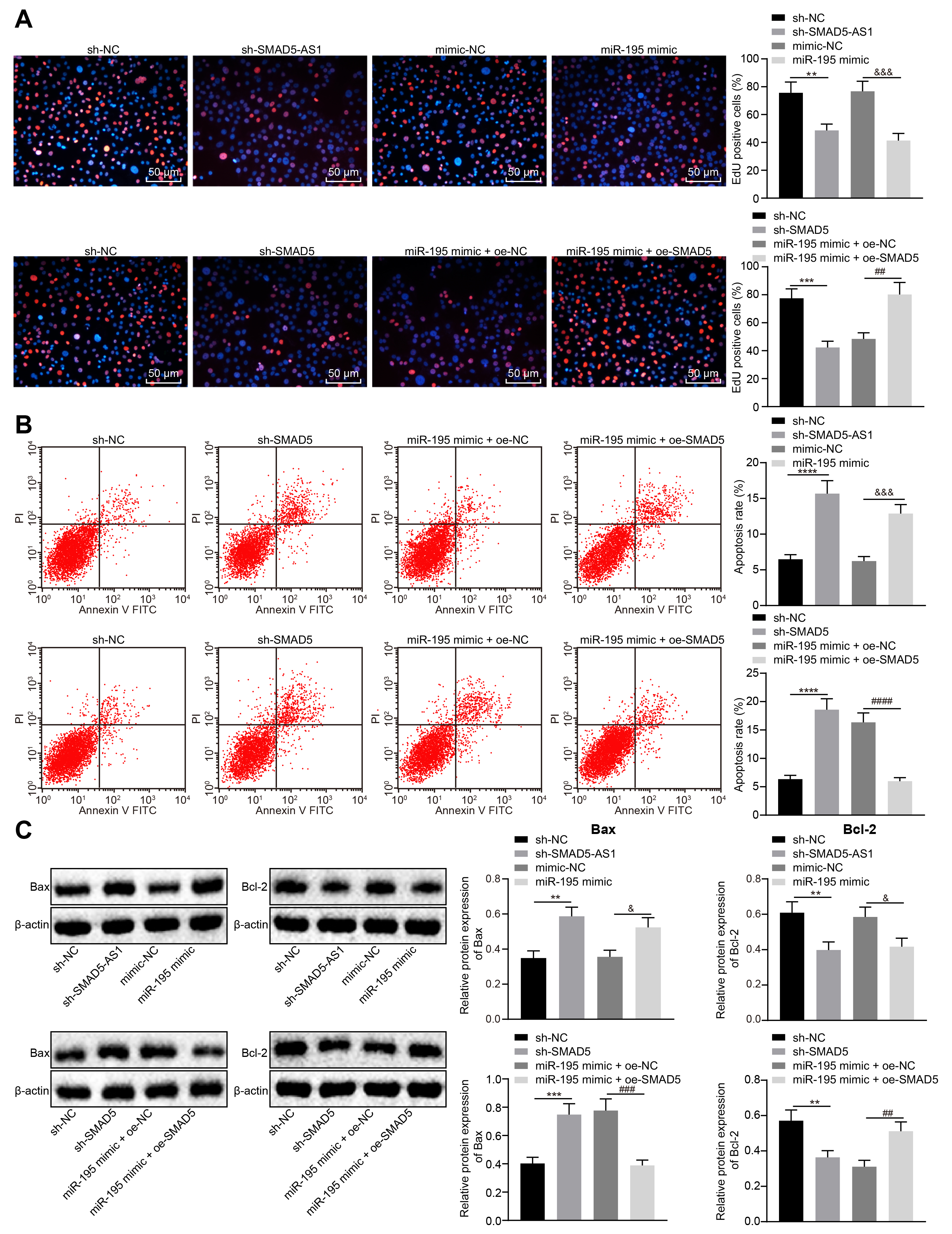

Supplement: Supplementary Figure 3 — SMAD5-AS1 and SMAD5 silencing or miR-195 overexpression impeded CNE-1 cell proliferation and enhances cell apoptosis. (A) CNE-1 cell proliferation assessed by EdU assay (200 ×, scale bar = 50 um). ***p < 0.001 vs. the sh-NC group; **p < 0.01 vs. the sh-NC group; &&&p < 0.001 vs. the mimic-NC group; ##p < 0.01 vs. the miR-195 mimic + oe-NC group. (B) CNE-1 cell apoptosis rate measured using flow cytometry. ****p < 0.0001 vs. the sh-NC group; &&&p < 0.001 vs. the mimic-NC group; ####p < 0.0001 vs. the miR-195 mimic + oe-NC group. (C) The protein expression of apoptosis-related factors Bax and Bcl-2 in CNE-1 cells determined by western blot analysis. ***p < 0.001 vs. the sh-NC group; **p < 0.01 vs. the sh-NC group; &p < 0.05 vs. the mimic-NC group; ###p < 0.001 vs. the miR-195 mimic + oe-NC group; ##p < 0.01 vs. the miR-195 mimic + oe-NC group. The measurement data were depicted as mean ± standard deviation. Data between two groups were tested using independent sample t-test. The experiment was repeated 3 times. [file Image_3.jpg]

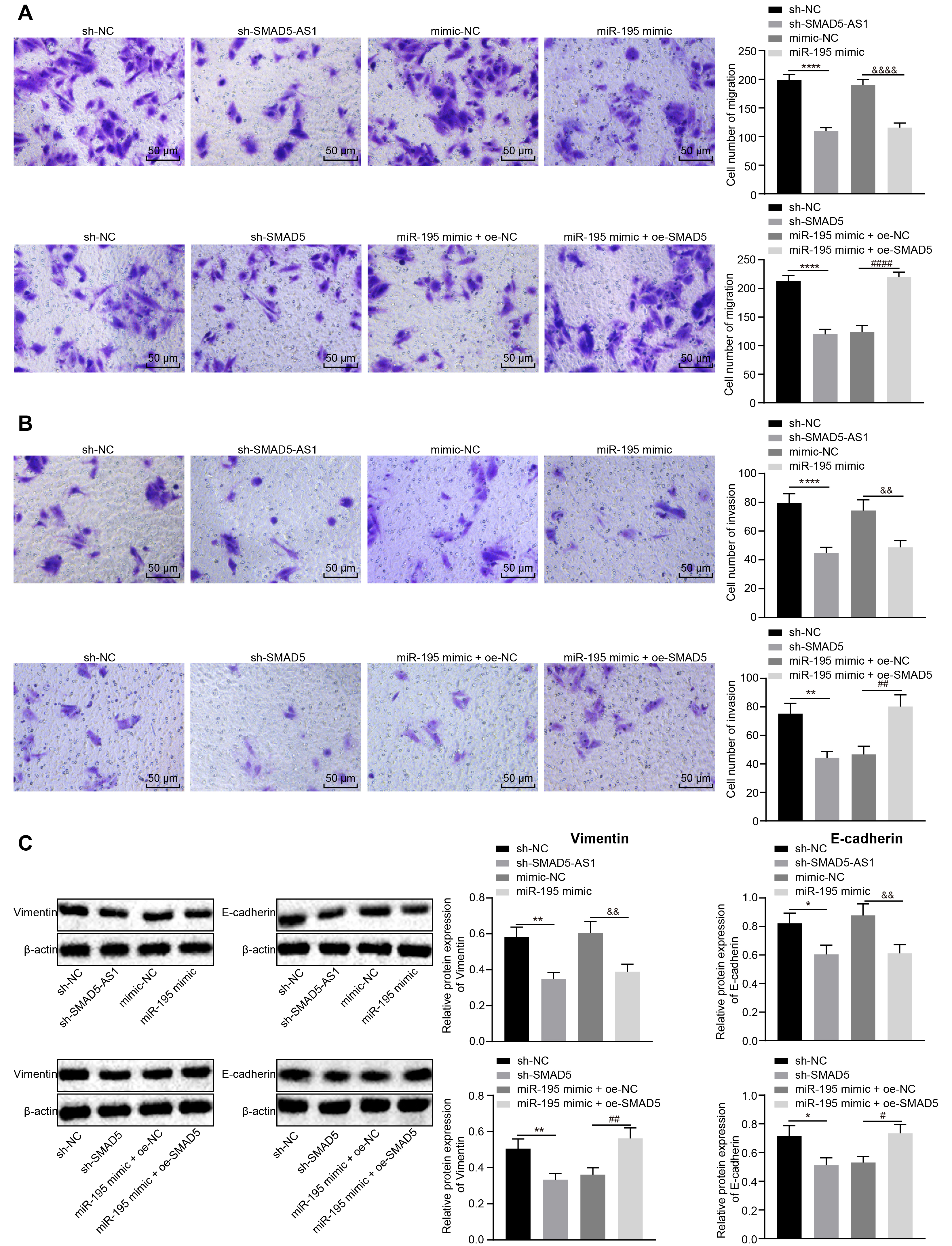

Supplement: Supplementary Figure 4 — SMAD5-AS1 and SMAD5 silencing or miR-195 overexpression disrupted CNE-1 cell invasion, migration and reversed EMT. (A) CNE-1 cell migration measured by Transwell assay (200 ×, scale bar = 50 um). ****p < 0.0001 vs. the sh-NC group; &&&&p < 0.0001 vs. the mimic-NC group; ####p < 0.0001 vs. the miR-195 mimic + oe-NC group. (B) CNE-1 cell invasion measured by Transwell assay (200 ×, scale bar = 50 um). ****p < 0.0001 vs. the sh-NC group; **p < 0.01 vs. the sh-NC group; &&p < 0.01 vs. the mimic-NC group; ##p < 0.01 vs. the miR-195 mimic + oe-NC group. (C) The protein expression of Vimentin and E-cadherin in CNE-1 cells determined by western blot analysis, **p < 0.01 vs. the sh-NC group; *p < 0.05 vs. the sh-NC group; &&p < 0.01 vs. the mimic-NC group; ##p < 0.01 vs. the miR-195 mimic + oe-NC group; #p < 0.05 vs. the miR-195 mimic + oe-NC group. The measurement data were expressed as mean ± standard deviation. n = 6. Independent sample t-test was adopted to compare data between two groups. The experiment was repeated 3 times. [file Image_4.jpg]

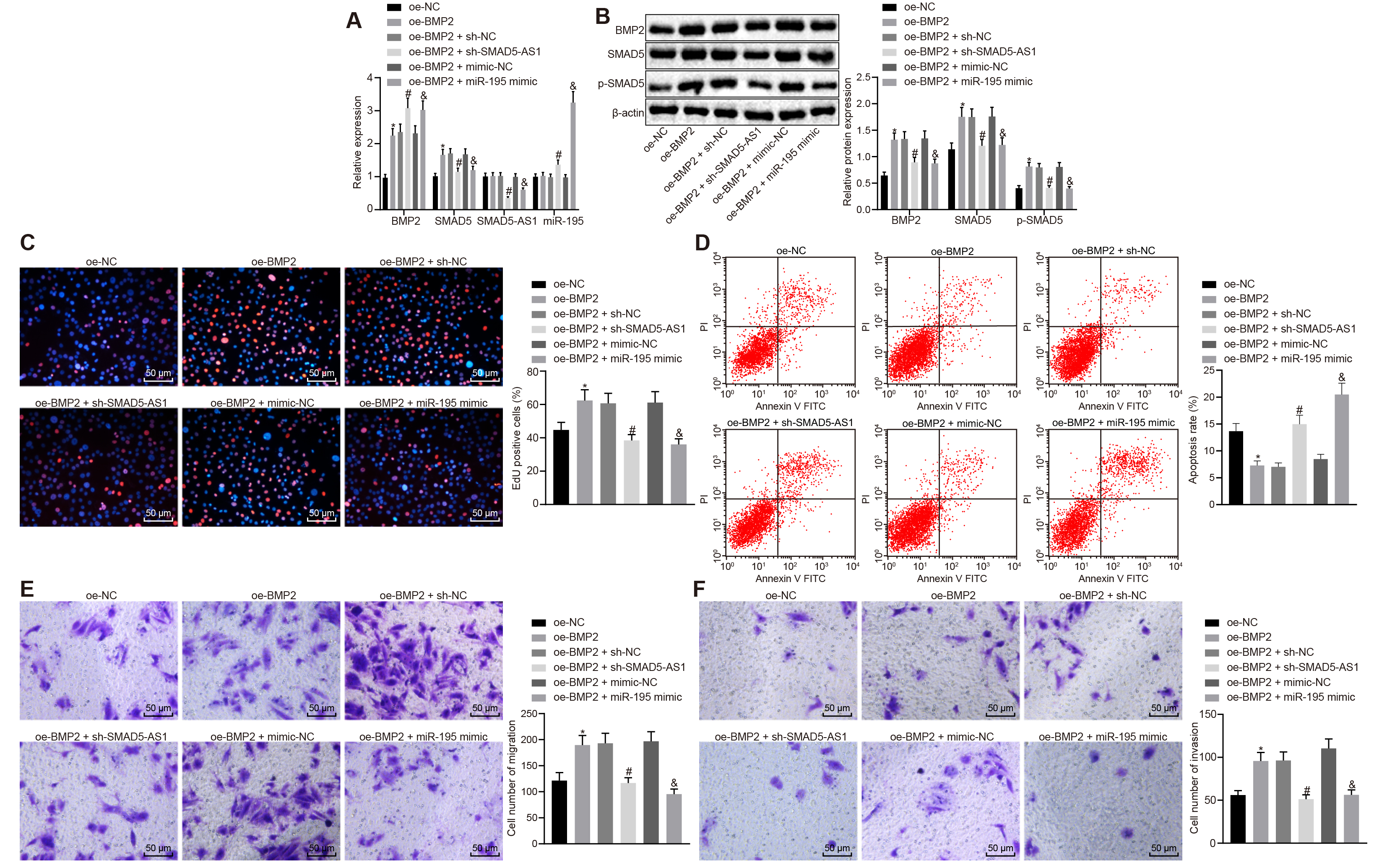

Supplement: Supplementary Figure 5 — Silencing of SMAD5-AS1 or miR-195 overexpression impeded CNE-1 cell growth and migration via blocking the BMP2/SMAD5 pathway. (A) The expression of BMP2, miR-195, SMAD5, and SMAD5-AS1 in CNE-1 cells determined using RT-qPCR. (B) The protein expression of BMP2 and phosphorylated SMAD5 in CNE-1 cells measured using western blot analysis. (C) CNE-1 cell proliferation assessed by EdU assay. (D) CNE-1 cell apoptosis rate measured by flow cytometry. (E) CNE-1 cell migration measured by Transwell assay (200 ×, scale bar = 50 um). (F) CNE-1 cell invasion measured by Transwell assay (200 ×, scale bar = 50 um). * vs. the oe-NC group, #p < 0.05 vs. the oe-BMP2 + sh-NC group, &p < 0.05 vs. the oe-BMP2 + mimic-NC group. The measurement data were expressed as mean ± standard deviation. n = 6. Independent sample t-test was adopted to compare data between two groups. The experiment was repeated 3 times. [file Image_5.JPEG]
